# Antigen-Specific B Cells Reactivate an Effective Cytotoxic T Cell Response against Phagocytosed *Salmonella* through Cross-Presentation

**DOI:** 10.1371/journal.pone.0013016

**Published:** 2010-09-27

**Authors:** Jelle de Wit, Yuri Souwer, Tineke Jorritsma, Hanny Klaasse Bos, Anja ten Brinke, Jacques Neefjes, S. Marieke van Ham

**Affiliations:** 1 Sanquin Research and Landsteiner Laboratory, Department of Immunopathology, Academic Medical Center, University of Amsterdam, Amsterdam, The Netherlands; 2 Division of Cell Biology, The Netherlands Cancer Institute, Amsterdam, The Netherlands; New York University, United States of America

## Abstract

**Background:**

The eradication of facultative intracellular bacterial pathogens, like *Salmonella typhi*, requires the concerted action of both the humoral immune response and the cytotoxic CD8^+^ T cell response. Dendritic cells (DCs) are considered to orchestrate the cytotoxic CD8^+^ T cell response via cross-presentation of bacterial antigens onto MHC class I molecules. Cross-presentation of *Salmonella* by DCs however, is accompanied by the induction of apoptosis in the DCs. Besides antibody production, B cells are required to clear *Salmonella* infection for other unknown reasons.

**Methodology/Principal Findings:**

Here we show that *Salmonella*-specific B cells that phagocytose *Salmonella* upon BCR-ligation reactivate human memory CD8^+^ T cells via cross-presentation yielding a *Salmonella*-specific cytotoxic T cell response. The reactivation of CD8^+^ T cells is dependent on CD4^+^ T cell help. Unlike the DCs, B cell-mediated cross-presentation of *Salmonella* does not coincide with apoptosis.

**Conclusions/Significance:**

B cells form a new player in the activation of the cytotoxic effector arm of the immune response and the generation of effective adaptive immunity in *Salmonella* infection.

## Introduction

*Salmonella* is a pathogenic bacterium that causes severe disease in mice and man. *Salmonella typhi* (*Salmonella enterica* serovar Typhi) causes invasive diseases in human, which has many features in common with *Salmonella typhimurium* in mice. The gastrointestinal tract is the major site of primary infection of the host and has to be passed before systemic infection can occur. One way to infect the host cells is via sampling of bacteria by DCs in the intestine. *In vitro* studies showed that DCs located in the lamina propria under the gut epithelium of the small bowel extend processes across the tight junctions between the epithelial cells and capture bacteria from the luminal side of the gut [1], [2]. The major route of infection however, is via microfold cells or M cells [3], [4]. The specialized antigen-sampling M cells are located in the dome region of the Peyer's Patches and are efficient in transportation of macromolecules and microorganisms to the underlying immune cells [2], [5]. Like other Gram-negative bacteria, *Salmonella* uses specific virulence factors to invade other cell types, called the Type III Secretion System (TTSS). Many *Salmonella* virulence genes are clustered in *Salmonella* pathogenicity islands (SPIs). SPI-1 and SPI-2 encode TTSSs that mediate the injection of effector proteins into the host cell cytoplasm via sophisticated secretion devices [6]. SPI-1 is associated with invasion of intestinal epithelia and enhanced intestinal inflammation in the infected host [7], [8]. SPI-2 modulates intracellular trafficking and enables replication within a modified vacuolar compartment, called the *Salmonella*-containing vacuole (SCV) [9]–[11] and enhances inflammation during enteric phase [12], [13]. *Salmonella* activates the PKB/Akt1 pathway to prevent maturation of SCV into destructive phagolysosomes, thus manipulating the host for its own survival [14].

After transcytosis by M cells, *Salmonella* reaches the subepithelial dome of the Peyer's patches and encounters an extensive network of resident macrophages, DCs and great numbers of B cells [15], [16]. Instead of being immediately destroyed by these cells, *Salmonella* have evolved several mechanisms to survive in the harsh milieu of phagosomal compartments [17] and can be cytotoxic to macrophages by inducing apoptosis *in vitro* [18], [19].

Recently, we showed that recognition of *Salmonella* via the specific B cell receptor (BCR) on B cells results in internalization of *Salmonella*. *Salmonella* is able to survive intracellularly in primary B cells in a non-replicative state [20]. Following uptake of *Salmonella*, B cells do not go into apoptosis, but differentiate and start to produce *Salmonella*-specific antibodies. In addition, BCR-mediated phagocytosis of *Salmonella* by B cells leads to antigen presentation via MHC class II and subsequent CD4^+^ T cell activation, which in turn boosts antibody production by the infected B cell.

Antibody transfer studies have shown that the requirement for B cells in the clearance of *Salmonella* does not solely depend on antibody formation [21]. Which additional immune responses need B cell involvement remains unclear. For clearance of *Salmonella*, not only the humoral immune response is required, but also the activation of cytotoxic CD8^+^ T cells is needed to eliminate *Salmonella*-infected cells. Recently, DCs have been shown to activate *Salmonella*-specific CD8^+^ memory T cells after direct uptake of bacteria or via suicide cross-presentation after uptake of *S. typhi*-infected human cells [22]. As the generation of *Salmonella* antigens for MHC class II molecules is an efficient process in infected B cells, we tested whether BCR-mediated phagocytosis also leads to cross-presentation of *Salmonella* antigens via the MHC class I pathway of B cells and whether this elicits a cytotoxic T cell response against *Salmonella*-infected cells.

Here we show that *Salmonella*-specific primary B cells that have internalized *Salmonella* do cross-present *Salmonella* antigens via MHC class I in a proteasome-dependent manner. Cross-presentation of *Salmonella* antigens by B cells reactivates *Salmonella*-specific CD8^+^ memory cells that acquire a cytotoxic phenotype and are efficient in killing of *Salmonella*-infected cells. Thus, antigen-specific B cells are an under appreciated type of cell for the induction of a cytotoxic T cell response against facultative intracellular bacteria.

## Results

### *Salmonella*-infected B Cells Initiate a CD8^+^ T Cell Response

**Figure 1:**
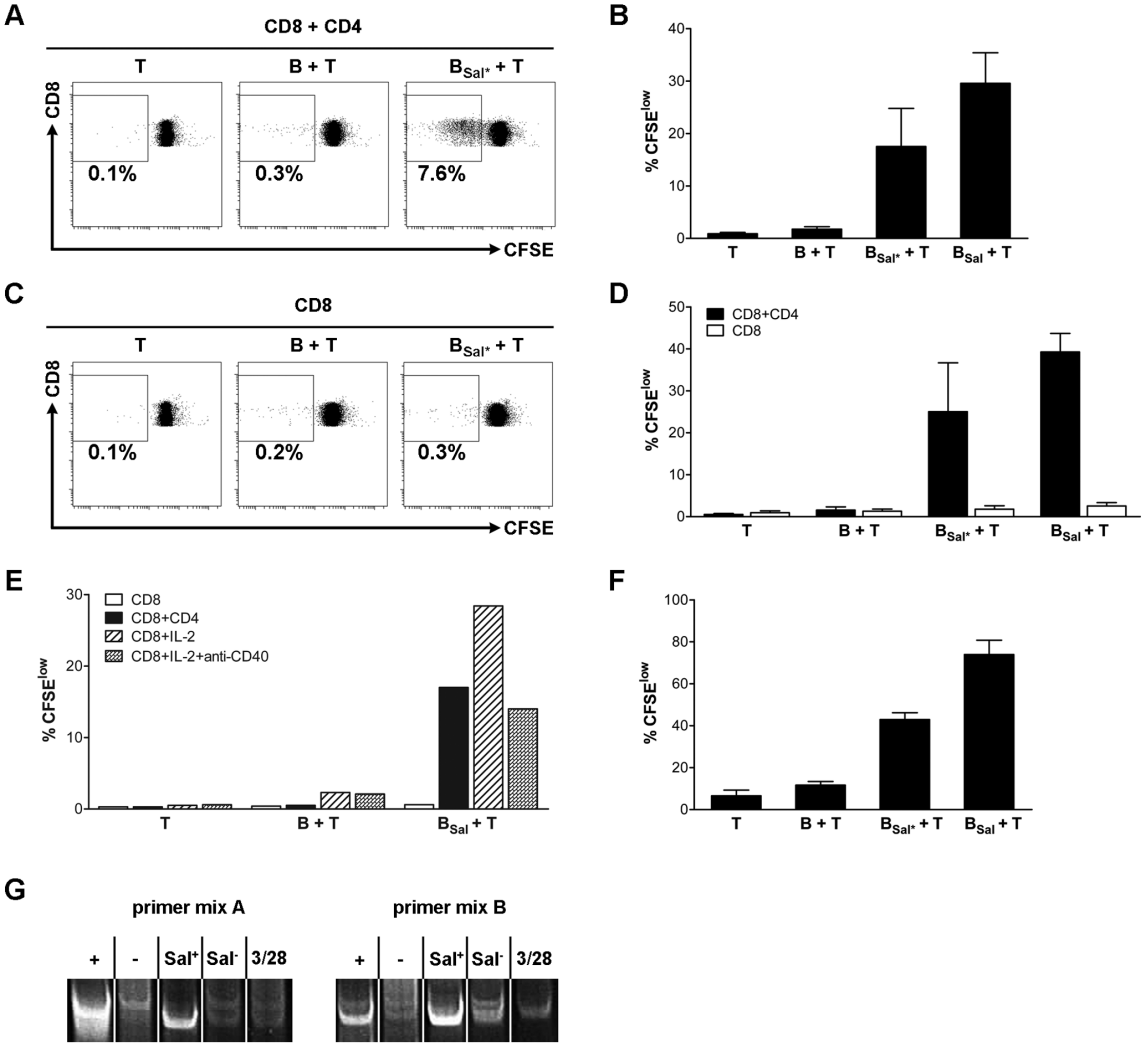
*Salmonella*-infected B cells induce CD8^+^ T cell proliferation with help of CD4^+^ T cells. (A) CFSE labeled CD8^+^ T cells were cultured alone (T), together with B cells (B + T) or together with B cells that had naturally phagocytosed *Salmonella* (B_Sal*_ + T), in the presence of CD4^+^ T cells. Proliferation was measured after 6 days. (B) CD8^+^ T cell proliferation was measured after culture with B cells that had naturally phagocytosed *Salmonella* (B_Sal*_) or anti-IgM-coated *Salmonella* (B_Sal_), in presence of CD4^+^ T cell help. The data are expressed as mean ± SEM, of seven independent experiments of different donors. (C) Proliferation of CFSE labeled CD8^+^ T cells (shown in A) in absence of CD4^+^ T cells. (D) CD8^+^ proliferation after culture with B cells that have naturally phagocytosed (B_Sal*_) or coated (B_Sal_) *Salmonella*, in the presence of CD4^+^ T cell help (black bars) or not (open bars). The data are expressed as mean ± SEM, of four independent experiments of different donors. (E) CD8^+^ T cells were cultured with B cells that had phagocytosed anti-IgM coated *Salmonella* (B_Sal_), in absence of help (open bars), in presence of CD4^+^ T cell help (black bars), in addition of IL-2 (striped bars), or in addition of IL-2 plus anti-CD40 antibodies. Data shown are from one representative experiment of three independent experiments with different donors. (F) CD8^+^ T cells were primed with B cells that had naturally phagocytosed S*almonella* and the proliferating CD8^+^ T cells were sorted after 6 days, expanded with IL-2. Resting CD8^+^ T cells were again CFSE labeled at day 13 and restimulated with autologous B cells or B cells that had phagocytosed *Salmonella* naturally (B_Sal*_) or coated *Salmonella* (B_Sal_), in presence of IL-2 (50 IU/ml). The data are expressed as mean ± SEM, of two independent experiments of different donors. (G) CD8^+^ T cells were activated with B cells that had naturally phagocytosed *Salmonella*, or non-specific via anti-CD3 and anti-CD28 antibodies. CD8^+^ T cells were sorted after 6 days and TCR clonality was measured. Shown are two different V-J primer mixes, with a positive control (+), negative control (−), sorted proliferating CD8^+^ T cells upon infected B cells (Sal^+^), sorted non-proliferating CD8^+^ T cells upon infected B cells (Sal^−^) and non-specific stimulated CD8^+^ T cells (3/28). Data are of one experiment.

**Figure 2:**
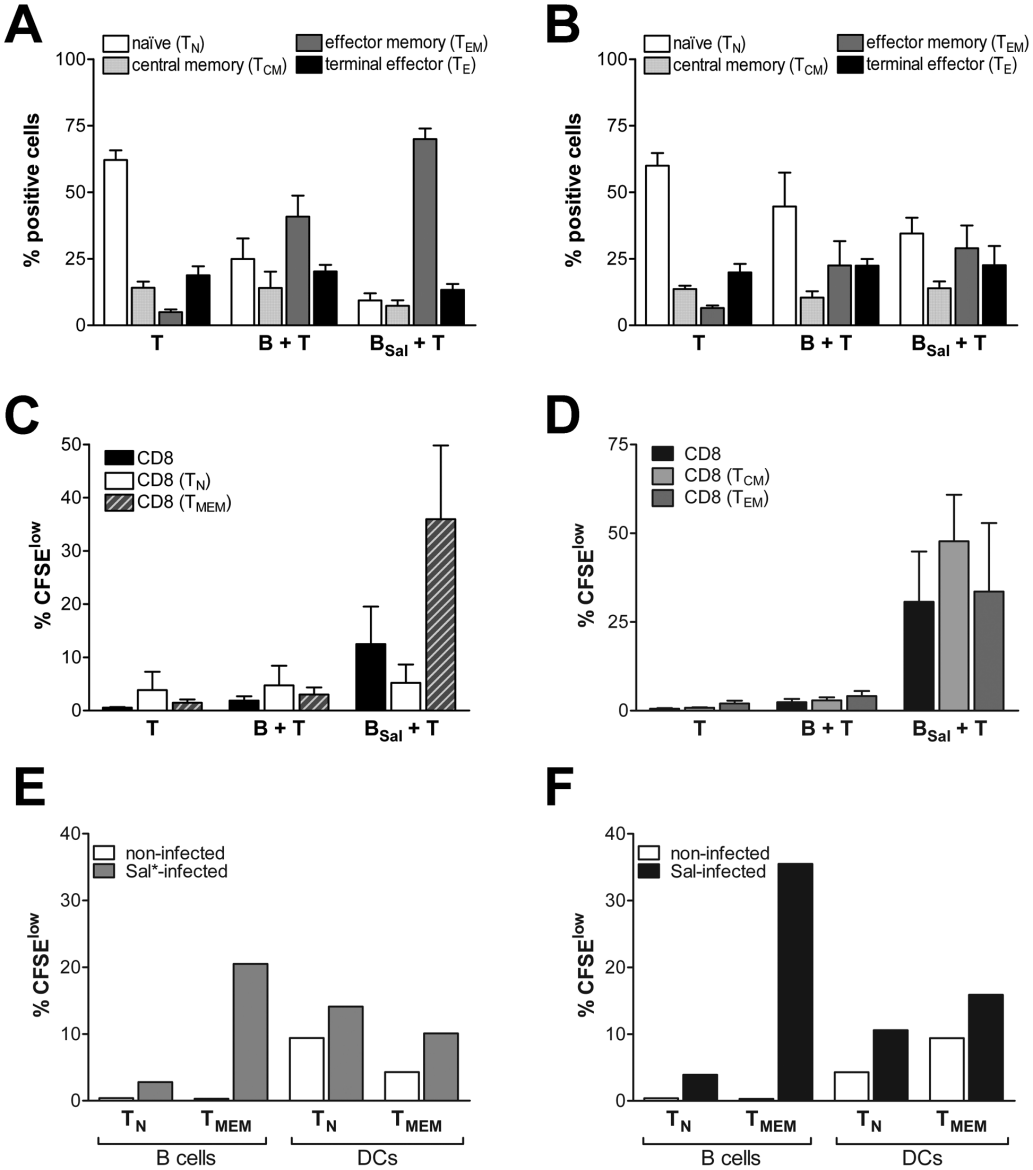
*Salmonella*-infected B cells reactivate a CD8^+^ memory response. (A) CD4^+^ and CD8^+^ T cells were cultured alone (T), together with B cells (B + T) or with anti-IgM-coated *Salmonella*-infected B cells (B_Sal_ + T). After 11 days, cells were stained for CD45RO and CD27 to discriminate between the different T cell populations: naïve (T_N_; CD45RA^+^CD27^+^), central memory (T_CM_; CD45RO^+^CD27^+^), effector memory (T_EM_; CD45RO^+^CD27^−^) and terminal effector (T_E_; CD45RA^+^CD27^−^) T cells. Gated CD8^+^ cells were analyzed. (B) Without CD4^+^ T cells, the differentiation of CD8^+^ T cells to effector memory is attenuated. Data are mean ± SEM from five independent experiments. (C) Coated *Salmonella*-infected B cells activate proliferation of sorted CD8^+^CD45RO^+^ cells (T_MEM_), but not of purified CD8^+^CD45RA^+^ cells (T_N_) in the presence of CD4^+^ help. Data are mean ± SEM from four independent experiments. (D) Coated *Salmonella*-infected B cells activate proliferation of sorted CD8^+^CD45RO^+^CD27^+^ (T_CM_) and CD8^+^CD45RO^+^CD27^−^ (T_EM_) cells. Data are mean ± SEM from three independent experiments. (E) Sorted CD8^+^CD45RA^+^ (naïve, T_N_) or CD8^+^CD45RO^+^ (memory, T_MEM_) T cells were cocultured with B cells or DCs, naturally infected with *Salmonella* (Sal*), in presence of CD4^+^ T cell help. Proliferation of the naïve or memory CD8^+^ T cells was measured at day 6. Data shown are from one representative experiment of two independent experiments with different donors. (F) Sorted naïve or memory CD8^+^ T cells were cocultured with B cells or DCs, infected with coated *Salmonella* (Sal), in presence of CD4^+^ T cell help. Proliferation of the naïve or memory CD8^+^ T cells was measured at day 6. Data shown are from one representative experiment of two independent experiments with different donors.

To study MHC class I antigen presentation by B cells, we used *Salmonella typhimurium* as a model for cross-presentation against facultative intracellular bacteria. Previously, we showed that about 4% of the B cells recognize *Salmonella* by their BCR, phagocytose *Salmonella*, and subsequently initiate a CD4^+^ T cell response [20]. Now, we investigated whether *Salmonella*-infected B cells are also capable to induce a cytotoxic CD8^+^ T cell response. B cells were incubated with *Salmonella* to allow phagocytosis of the bacteria by B cells. After extensive washing, the *Salmonella*-infected B cells were co-cultured with CFSE-labeled CD4^+^ and CD8^+^ T cells. As observed before, B cells that had phagocytosed *Salmonella* induced CD4^+^ T cell proliferation [20]. Interestingly, a considerable amount of CD8^+^ T cells had proliferated as well (Fig. 1A and B). Since the amount of B cells that specifically recognize *Salmonella* via the BCR is quite low, we maximized the T cells responses by enhancing the uptake of *Salmonella* by B cells using *Salmonella* coated with a tetrameric antibody complex, consisting of anti-LPS antibodies and anti-IgM-BCR antibodies. As a result, all B cells expressing an IgM-BCR, recognize *Salmonella* and phagocytose the bacterium via their BCR. This resulted in an uptake of *Salmonella* by 30% to 60% of the B cells (data not shown) and a strong increase in CD8^+^ T cell proliferation in B/T co-culture experiments. Next, we investigated the requirement of CD4^+^ T cell help for the proliferation of the CD8^+^ T cells. *Salmonella*-infected B cells were cultured with CD8^+^ T cells in the absence of CD4^+^ T cells. This situation almost completely abolished proliferation of the CD8^+^ T cells with both naturally phagocytosed *Salmonella*-infected B cells or coated *Salmonella*-infected B cells (Fig. 1C and D). Thus, B cells infected by *Salmonella* act as antigen presenting cells and induce CD8^+^ T cell proliferation, but activation of CD8^+^ T cells requires the simultaneous CD4^+^ T cell activation to enable T cell help. To study which kind of help CD4^+^ T cells provide for CD8^+^ T cell proliferation, we looked at the requirement of IL-2, by adding blocking antibodies to the culture of infected B cells and CD4^+^ and CD8^+^ T cells. This resulted in a very strong reduction of CD8^+^ T cell proliferation (Fig. S1). However, CD4^+^ T cell proliferation was also decreased (data not shown). Therefore we performed a converse experiment, by adding IL-2 instead of CD4^+^ cells to the co-culture of infected B cells and CD8^+^ T cells. This shows that substitution of CD4^+^ T cell help by IL-2 also induces CD8^+^ T cell proliferation by B cells that have phagocytosed *Salmonella* (Fig. 1E). The addition of IL-2 to the co-culture with non-infected B cells did not induce significant proliferation of CD8^+^ T cells, indicating that IL-2 itself does not stimulate non-specific CD8^+^ T cell proliferation. Furthermore, the role of CD40 stimulation was studied. Since CD40-CD40L interactions do also play a role in CD4^+^ T cell activation, CD4^+^ T cell help was substituted by IL-2. Blocking CD40 resulted in reduction of CD8^+^ T cell proliferation (Fig. 2E). Thus CD4^+^ T cells help is partially mediated by both IL-2 and CD40-CD40L interactions.

Although CD8^+^ T cells proliferate only in presence *Salmonella*-infected B cells and CD4^+^ T cell help, we cannot exclude that the CD8^+^ T cell response is partly an effect of bystander proliferation, for instance via soluble factors. Therefore, we studied the T cell response in more detail. Culture supernatant of infected B cells in presence of CD4^+^ T cell help was not able to induce CD8^+^ T cell proliferation (data not shown). In addition, blocking experiments, in which CD8^+^ T cell activation was prevented via anti-CD8 blocking antibodies, resulted in a decrease of CD8^+^ T cell activation by *Salmonella*-infected B cells (Fig. S1). To study *Salmonella*-specific T cell proliferation in more detail, restimulation of the T cells was studied. Proliferating CD8^+^ T cells which respond to *Salmonella*-infected B cells were sorted. These *Salmonella*-responsive T cells were restimulated with autologous B cells which were infected with *Salmonella*, or anti-IgM coated *Salmonella* to maximize the infection rate. During restimulation additional IL-2 was added for help, and CD8^+^ T cell proliferation was measured using CFSE labeling. This showed that CD8^+^ T cells are primary *Salmonella*-specific, because they proliferate mainly upon restimulation with *Salmonella*-infected B cells (Fig. 1F). In addition, we analyzed clonality of the *Salmonella*-specific T cell response using a TCR beta clonality assay, as an additional indication for antigen-specific T cell activation. Proliferating CD8^+^ T cells, which were activated by S*almonella*-infected B cells, and non-proliferating CD8^+^ T cells, which did not respond, were both sorted and tested for clonal TCR beta gene rearrangements. Subsequently, CD8^+^ T cells were stimulated with anti-CD3 and anti-CD28 antibodies to obtain non-specific proliferated T cells. The CD8^+^ T cells which proliferate upon *Salmonella*-infected B cells show a clear clonality (Fig. 1G). In contrast, neither CD8^+^ T cells did proliferate after culturing with infected B cells, nor non-specific CD8^+^ T cells proliferating cells show a clear TCR clonality. Thus, primary B cells which have phagocytosed *Salmonella* elicit CD8^+^ T cell proliferation, which is mainly *Salmonella*-specific.

### *Salmonella*-infected B Cells Activate Both the Central Memory and Effector Memory CD8^+^ Compartment

Do B cells elicit a naïve or a memory CD8^+^ cell response? To study this, we cultured the total CD8^+^ T cell population with or without *Salmonella*-infected B cells, in presence of CD4^+^ T cells for help, and determined the naïve (CD45RO^−^CD27^+^; T_N_), central memory (CD45RO^+^CD27^+^; T_CM_), effector memory (CD45RO^+^CD27^−^; T_EM_) or terminal effector (CD45RO^−^CD27^−^; T_E_) phenotype of the CD8^+^ T cells after prolonged culture. Upon 11 days of culture, purified CD8^+^ T cells show mainly a naïve phenotype. In contrast, following activation with *Salmonella*-infected B cells, the CD8^+^ population shifts towards the T_EM_ phenotype (Fig. 2A). CD8^+^ T cells that are activated with *Salmonella*-infected B cells in the absence of CD4^+^ T help, do not differentiate to a T_EM_ phenotype (Fig. 2B). Next, we investigated if *Salmonella*-infected B cells can prime naïve T cells at all. Therefore we studied proliferation of sorted naïve (CD45RA^+^CD45RO^−^; T_N_) or memory (CD45RA^−^CD45RO^+^; T_MEM_) CD8^+^ T cells in response to *Salmonella*-infected B cells. Figure 2C illustrates that the naïve CD8^+^ population do not proliferate over background values upon stimulation with *Salmonella*-infected B cells, whereas the memory CD8^+^ T cells proliferated vigorously. Further discrimination of the memory subset by sorting T_CM_ (CD45RO^+^CD27^+^) and T_EM_ (CD45RO^+^CD27^−^) CD8^+^ T cells, followed by co-culture with *Salmonella*-infected B cells, showed that both T_CM_ and T_EM_ can be activated by *Salmonella*-infected B cells, although that T_EM_ proliferate less compared to T_CM_ (Fig. 2D). Recent experiments in mice show that both T_CM_ and T_EM_ cells can arise from activation and proliferation of the T_CM_ compartment, whereas T_EM_ cells are more terminally differentiated and therefore proliferate poorly and only give rise to T_EM_ progeny [23]. Indeed, expansion of the sorted T_CM_ by *Salmonella*-infected B cells yielded offspring with both a T_CM_ and a T_EM_ phenotype, whereas T_EM_ activation yielded mainly T_EM_ progeny (data not shown). In addition, the effectiveness of T cell activation by infected B cells was compared using another antigen presenting cell, a dendritic cell (DC). After uptake of *Salmonella*, the DCs matured (data not shown) and were capable to activate both naïve (T_N_) and memory CD8^+^ T cells (T_MEM_), whereas B cells could only reactivate memory CD8^+^ T cells (Fig. 2E). However, the percentage of B cells that had taken up *Salmonella* was much lower compared to the DCs that had taken up *Salmonella* (Fig. S2). To correct for these differences in uptake, we also used anti-IgM coated *Salmonella*, to enhance the number of *Salmonella*-infected B cells. Indeed this yielded comparable amounts of *Salmonella*-infected B cells and DCs (Fig. S2). In this setting, *Salmonella*-infected B cells were even superior in activation of memory CD8^+^ cells (Fig. 2F). So, upon *Salmonella*-infection, B cells can reactive CD8^+^ memory cells in the same manner as DCs. In summary, these data show that *Salmonella*-infected B cells activate an effective recall response of memory CD8^+^ T cells, yielding expansion of both the “memory stem cell” containing T_CM_ compartment and the memory effector cell containing T_EM_ compartments.

### Activated *Salmonella*-specific CTLs are Able to Kill *Salmonella*-infected Cells

**Figure 3:**
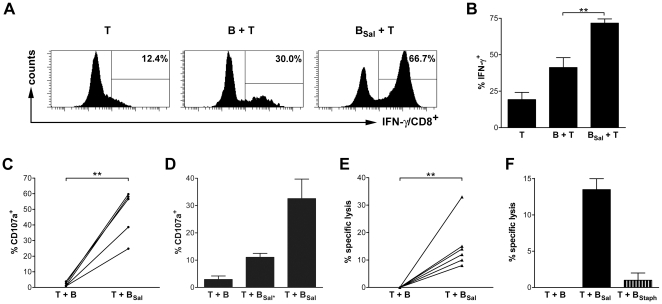
*Salmonella-*infected B cells induce *Salmonella*-specific CD8^+^ T cells to secrete IFN-γ and are cytotoxic. (A) CD4^+^ and CD8^+^ T cells were cultured alone (T), with B cells (B + T) or with anti-IgM-coated *Salmonella*-infected B cells (B_Sal_ + T). After 11 days, CD8^+^ T cells were stimulated for 5 hours with PMA, ionomycin and BFA and analyzed for IFN-γ production by intracellular FACS staining. A representative experiment of five independent experiments using cells from different healthy donors is shown. (B) *Salmonella*-infected B cells induce IFN-γ-expression by CD8^+^ T cells compared to non-infected B cells. Data are the mean ± SEM from five independent experiments of different donors and ** p<0.01. (C) CD8^+^ T cells were primed with coated *Salmonella*-infected B cells and the proliferating CD8^+^ T cells were sorted after 6 days, expanded with IL-2 for another 6 days and restimulated with autologous B cells that had either or not phagocytosed *Salmonella*. *Salmonella*-specific degranulation was measured by CD107a expression at the plasma membrane CD8^+^ T cells. Data are means ± SEM of five independent experiments of different donors, and ** p<0.01. (D) Proliferating CD8^+^ T cells primed with B cells that had naturally phagocytosed *Salmonella* were sorted and CD107a expression was measured upon re-encounter of B cells (T + B), B cells naturally infected with *Salmonella* (T + B_Sal*_) or anti-IgM-coated *Salmonella* (T + B_Sal_). Data are of two experiments. (E) Sorted *Salmonella*-primed CD8^+^ T cells (see C) specifically kill *Salmonella*-infected B cells as measured by ^51^Cr release of autologous B cells that were either or not infected with *Salmonella.* Data are expressed as mean ± SEM, of six independent experiments of different donors, and ** p<0.01. (F) Sorted *Salmonella*-primed CD8^+^ T cells (see C) do not kill *Staphylococci*-infected B cells as measured by ^51^Cr release of autologous B cells that were either infected with *Salmonella* or with *Staphylococci*. The data are expressed as mean ± SEM, of two independent experiments of different donors.

Activation of cytotoxic CD8^+^ T cells by B cells is controversial. Earlier reports showed that B cells induce tolerance or anergy in CD8^+^ T cells. In contrast, we showed proliferation of CD8^+^ T cells upon activation by antigen-specific B cell upon phagocytosis of *Salmonella*. Although this proliferation is unlikely to yield tolerance, proliferation itself does not guarantee that the activated CD8^+^ T cells have acquired a functional anti-bacterial phenotype. CD8^+^ T cells kill intracellular pathogens either via secretion of IFN-γ or via direct killing of the infected target cell [24]. To investigate if *Salmonella*-containing B cells induce a functional CD8^+^ T cell response, IFN-γ secretion of the activated CD8^+^ T cells was measured. This showed that, after culture with *Salmonella*-infected B cells, the number of CD8^+^ T cells producing IFN-γ is increased to more than 65% (Fig. 3A and 3B). Thus, cross-presentation of *Salmonella* antigens by B cells induces not only proliferation of the CD8^+^ T cells but also renders the cells functional in that it induces IFN-γ secretion.

As these data demonstrate that B cells that had taken up *Salmonella* are able to functionally activate CD8^+^ T cells, the question remained if the activated CD8^+^ T cells were *Salmonella*-specific and whether CD8^+^ T cells can acquire a cytotoxic phenotype through B cell-mediated activation. First, we investigated if the CD8^+^ T cells degranulate their cytotoxic granules upon recognition of *Salmonella*-infected target cells. For this we analyzed expression of the marker CD107a, which is expressed at the plasma membrane of CD8^+^ T cells upon degranulation. *Salmonella*-infected B cells were cultured together with CFSE labeled CD4^+^ and CD8^+^ T cells. The proliferating *Salmonella*-primed CD8^+^ T cells were sorted after 6 days, expanded and re-exposed to autologous B cells that had been infected to high percentages with anti-IgM-coated *Salmonella* after which CD107a expression was measured. Upon re-exposure to *Salmonella*-infected B cells, the *Salmonella*-primed CD8^+^ T cells show a strong and significant increase of CD107a expression at the plasma membrane. Since the proliferating CD8^+^ T cells did not show degranulation when re-exposed to autologous, non-infected B cells, *Salmonella*-primed CD8^+^ T cells degranulate specifically upon recognition of *Salmonella*-infected cells (Fig. 3C). To exclude the possibility that the anti-IgM coating of *Salmonella* had affected our results, we also primed CD8^+^ T cells with *Salmonella*-specific B cells that had naturally phagocytosed *Salmonella* via their antigen-specific BCR. These primed CD8^+^ T cells degranulated specifically upon recognition of autologous B cells naturally infected with *Salmonella*, of which a relative small percentage (5–10%, data not shown) contained *Salmonella*-infected antigen-specific B cells (Fig. 3D; middle bar). In addition, the naturally *Salmonella*-primed CD8^+^ T cells very efficiently degranulated upon contact with autologous B cells of which a higher percentage (26–46%, data not shown) of cells had been infected with anti-IgM coated *Salmonella* (Fig. 3*D*; right bar). Finally, we investigated if degranulation of the *Salmonella*-specific CD8^+^ T cells also leads to death of the *Salmonella*-infected cells to determine the true cytotoxic efficacy of the lytic granules. CFSE labeled CD8^+^ T cells were primed by incubation with *Salmonella*-infected B cells. Proliferating CD8^+^CFSE^low^ cells were sorted and expanded for another 6 days. Re-exposure of the *Salmonella*-primed T cells to *Salmonella*-infected, ^51^Cr-labeled autologous B cells demonstrated that the primed CD8^+^ T cells killed *Salmonella*-infected B cells but not non-infected B cells (Fig. 3E). In addition, the *Salmonella*-primed CD8^+^ T cells were not able to kill B cells that had phagocytosed *Staphyloccoci* via BCR ligation (Fig. 3F), demonstrating that the CD8^+^ cells are indeed at least partly *Salmonella*-specific and do not recognize autologous B cells that are activated via BCR-mediated uptake of other bacteria. Thus, activation of CD8^+^ T cells by B cells cross-presenting *Salmonella* antigens induces a cytotoxic phenotype in the CD8^+^ T cells that specifically mediates killing of *Salmonella*-infected cells.

### Cross-Presentation of *Salmonella*-antigens is Partly Proteasome Dependent

**Figure 4:**
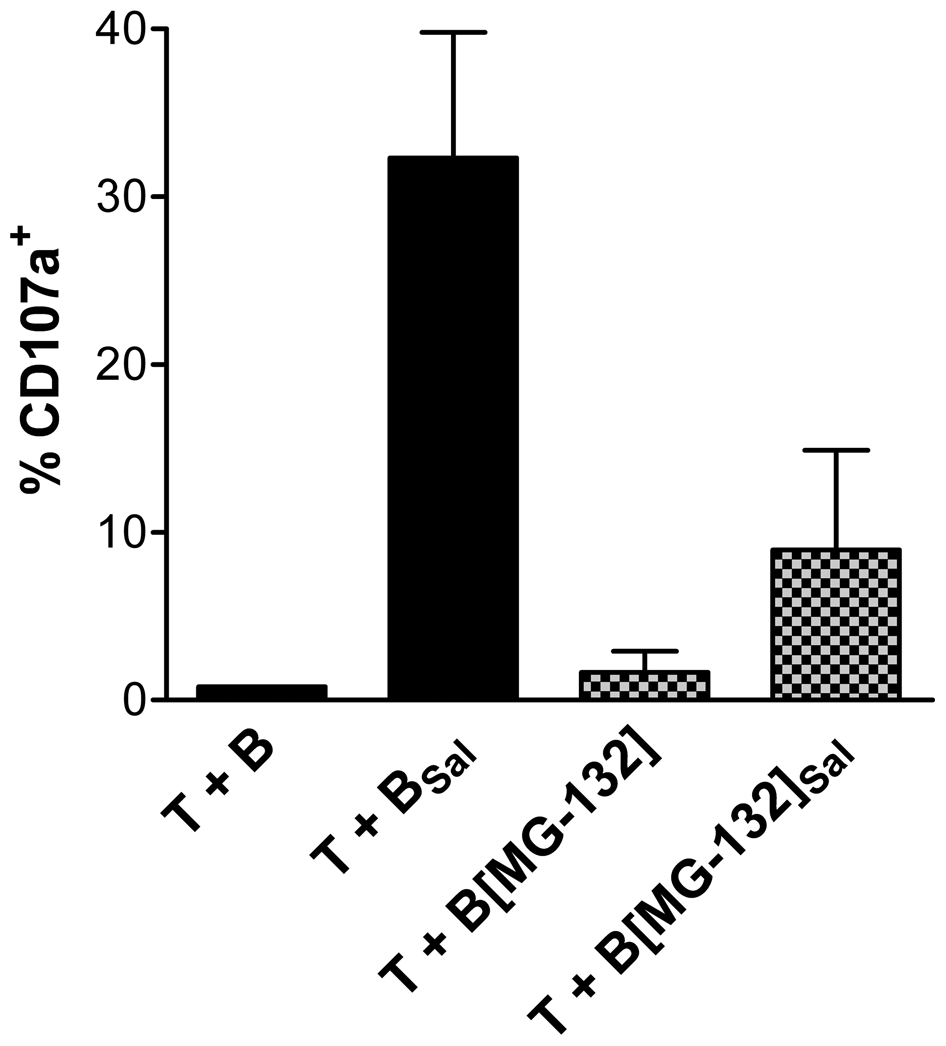
Proteasome inhibition of *Salmonella*-infected B cells diminishes degranulation of *Salmonella*-specific CD8^+^ T cells. CD8^+^ T cells were activated by *Salmonella*-infected B cells and after 6 days, *Salmonella*-specific, proliferating CD8^+^ T cells were sorted. Next, the sorted CD8+ T cells were reactivated by non-infected B cells (T + B) or *Salmonella*-infected B cells (T + B_Sal_). Pre-treatment of B cells with proteasome inhibitor MG-132 (20 µM) before reactivation leads to a decrease in degranulation of the *Salmonella*-specific CD8^+^ T cells (T + B[MG-132]_Sal_), as measured by CD107a expression. The data are expressed as mean ± SEM, of two independent experiments of different donors.

We showed that after phagocytosis of *Salmonella*, B cells are able to cross-present antigens to CD8^+^ T cells and thereby initiate a *Salmonella*-specific cytotoxic T cell response. The mechanism of cross-presentation of *Salmonella* antigens by B cells is unknown. One likely mechanism is that after phagocytosis, antigens are translocated from the *Salmonella*-containing vacuole (SCV) into the cytoplasm of B cells. Next, the antigens are degraded in the cytoplasm by proteasomes into small peptides, which are presented via the classical MHC class I antigen presentation route. To investigate this mechanism of cross-presentation, we used a chemical compound that specifically blocks the proteasome (MG-132). To study the effect of cross-presentation when blocking the proteasome, we used the CD107a degranulation assay. Re-exposure of *Salmonella*-primed CD8^+^ T cell to autologous, *Salmonella*-infected B cells showed that *Salmonella*-infected B cells are much less efficient in inducing CD8^+^ T cell degranulation when proteasomes are inhibited (Fig. 4). In addition, proteasome inhibition of the infected B cells also prevents killing by *Salmonella*-specific CD8^+^ T cells (data not shown). This observation implies that the proteasome is somehow involved in cross-presentation of *Salmonella* antigens by B cells. How the proteasome is involved is unclear as proteasome inhibition has many secondary effects on the ubiquitin cycle, the modification of histones and the formation of multivesicular bodies [25].

### B Cells do not Cross-Present Heat-killed *Salmonella*

**Figure 5:**
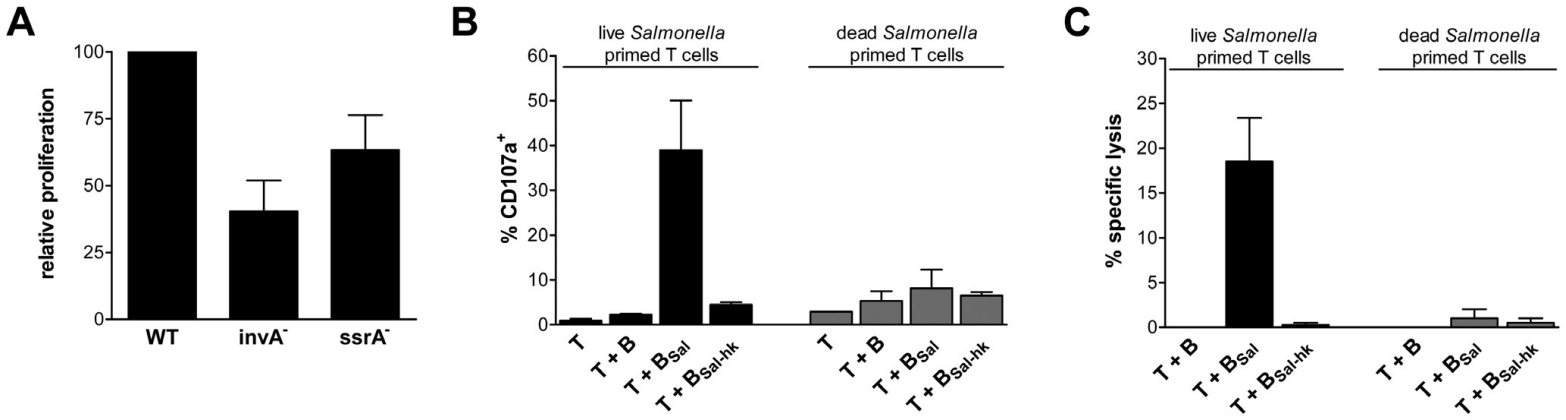
Induction of CTL response against *Salmonella* by B cells requires uptake of living *Salmonella*. (A) CD8^+^ T cells were labeled with CFSE and cultured with B cells that had phagocytosed either wild type *Salmonella* (WT), mutant for SPI-1 (invA^−^) or SPI-2 (ssrA^−^). Proliferation was measured after 6 days. Data shown are proliferation of CD8^+^ cells relative to wild type proliferation and are of two different donors; error bars are SEM. (B) *Salmonella*-specific T cells that were primed with B cells that had either internalized living (left panel) or dead *Salmonella* (right panel) were sorted and restimulated with B cells (T + B), B cells that had internalized living *Salmonella* (T + B_Sal_) or dead *Salmonella* (T + B_Sal-hk_). Degranulation was analyzed as CD107a expression by CD8^+^ T cells. (C) CD8^+^ T cell mediated kill was measured as the release of ^51^Cr by B cells. (B–C) Data are expressed as mean ± SEM, from four (live *Salmonella* primed) or two (dead *Salmonella* primed) independent experiments using material from different healthy donors.

*Salmonella* survives inside a cell via expression of the TTSS that create an intracellular environment that neutralizes the destructive forces of the host cell [26]. The TTSS components SPI-1 and SPI-2 play a role in this process by exporting proteins into the host cell. Because of the capacity of *Salmonella* to invade cells and to control its maintenance inside the cell via SPI-1 and SPI-2, it is possible that *Salmonella* itself might play a direct role in the cross-presentation pathway in B cells. To determine the role of SPI-1 and SPI-2 in cross-presentation of *Salmonella* by B cells, we analyzed CD8^+^ T cells proliferation via co-culture with CD4^+^ T cells and B cells that had phagocytosed anti-IgM-coated wild type *Salmonella*, or *Salmonella* with a mutation in SPI-1 (invA^−^) or SPI-2 (ssrA^−^). Both *Salmonella* mutants were still able to elicit a CD8^+^ T cell response, albeit at lower percentages of T cell proliferation compared to wild type *Salmonella* (Fig. 5A). Thus SPI-1 and SPI-2 each contribute to cross-presentation of *Salmonella* antigens by B cells, but are not essential.

By creating an environment in which *Salmonella* itself cannot be killed, it is possible that the intracellular survival plays a role in the efficacy of cross-presentation of *Salmonella* antigens by B cells. To study the contribution of *Salmonella* on the efficacy of cross-presentation of *Salmonella* antigens, we compared the efficiency of living and dead *Salmonella* to induce CTL activation. CD8^+^ T cells were primed with B cells infected with living or heat-killed *Salmonella*. After sorting and expansion, the *Salmonella*-primed T cells were re-exposed to autologous B cells that had either or not phagocytosed living or dead *Salmonella*. Cross-presentation of *Salmonella* antigen was measured by the extent of degranulation of the primed T cells using CD107a expression. CD8^+^ T cells that had been primed by B cells infected with living *Salmonella* efficiently degranulated upon recognition of autologous B cells that had phagocytosed living *Salmonella* (upregulation of CD107a to 60%), whereas the cells did not degranulate upon contact with B cells that had phagocytosed dead *Salmonella* (Fig. 5B; left panel). This indicates either that B cells present different *Salmonella* antigens to CD8^+^ T cells from live or dead bacteria or that B cells cannot cross-present *Salmonella* antigens when the intracellular bacterium is not alive. Furthermore, CD8^+^ T cells that had been primed with B cells that had taken up dead *Salmonella* showed poor degranulation to B cells that had taken up either live or heat-killed *Salmonella* (Fig. 5B; right panel), demonstrating that B cells indeed do not induce a cytotoxic CD8^+^ T cell response via cross-presentation of *Salmonella* antigens from dead intracellular bacteria. This observation was confirmed by studying elimination of *Salmonella*-infected B cells by ^51^Cr-release (Fig. 5C). Together, these results show that B cells are not able to cross-present antigens of dead *Salmonella* and that *Salmonella* contributes to the activation of a *Salmonella*-specific cytotoxic CD8^+^ memory response.

## Discussion

Studies in B-cell-deficient mice showed that protective immunity to *Salmonella* strongly depends on B cells [27]. This dependency does not only result from antibody generation, as passive transfer of *Salmonella*-immune serum cannot transfer resistance to *Salmonella* [28]. In addition, B cells are involved in the generation of a profound CD4^+^ and CD8^+^ T cell response after *Salmonella* infection [21], but the precise role of B cells remained unclear. We previously showed that human antigen-specific B cells that have internalized *Salmonella* via their BCR are able to induce a *Salmonella*-specific CD4^+^ T cell response which aids the humoral immune response against *Salmonella* [20]. The data described here may explain the role of B cells in the immune response against *Salmonella* infection other than antibody formation. In contrast to data in mouse B cell lines, in which uptake of *Salmonella* does not lead to antigen cross-presentation via MHC class I [29], we now demonstrate that *Salmonella*-specific human primary B cells that have phagocytosed *Salmonella* via their BCR are capable to induce a strong recall response of cytotoxic CD8^+^ T cells after cross-presentation of *Salmonella* antigens. We previously showed that in the human Ramos B cell line, *Salmonella* is not only capable to survive, but also to replicate intracellularly. In contrast, in primary human B cells *Salmonella* survives, but is unable to replicate inside the B cell [20]. A recent report shows that poor intracellular replication of *Salmonella* leads to reduced antigen presentation in DCs [30]. In contrast, we found that *Salmonella*-specific primary B cells are efficient in cross-presentation of *Salmonella* antigens from the non-replicating bacteria and activate *Salmonella*-specific CD8^+^ cells that show a functional cytotoxic T cell response. Thus, primary human B cells are capable in cross-presentation of non-replicating *Salmonella* to CD8^+^ T cells.

After internalization by the B cell, *Salmonella* survives in the SCV. For cross-presentation, *Salmonella*-antigens should be loaded onto MHC class I. The TTSS of *Salmonella* could play a role in delivery of antigens in the cytosol by injecting proteins directly into the cytosol [31]. These proteins can be degraded by the proteasome and after transportation into the ER, loaded onto MHC class I molecules. We demonstrated that both SPI-1 and SPI-2 contribute to cross-presentation, but are not individually required. It is therefore likely that antigens excreted in the host cytosol by SPI-1 and SPI-2 are both used for antigen processing to MHC class I. This is in line with the observation that B cells could not cross-present *Salmonella*-specific antigens of heat-killed *Salmonella* in which SPI-1 or SPI-2 are no longer active. Although the exact molecular pathways of cross-presentation in B cells remain to be elucidated, it is clear that *Salmonella* itself appears to be involved in the generation of an effective cytotoxic CD8^+^ T cell response against the bacteria upon B cell infection. This phenomenon points to the occurrence of co-evolution of bacterial immune evasion and the generation of effective anti-bacterial immunity.

Cross-presentation of *Salmonella* antigens by B cells leads to activation of a CD8^+^ cytotoxic T cell response, but help of CD4^+^ T cells is required. The main factor of CD4^+^ T cell help seems to be IL-2, since replacement of CD4^+^ T cell help by addition of IL-2 is sufficient to elicit CD8^+^ T cell proliferation, induced by *Salmonella* infection of the B cells. The CD4^+^ dependency of the CD8^+^ response led us to investigate if the observed responses were in fact mere bystander activation. Proliferation upon restimulation of *Salmonella*-primed CD8^+^ T cells implies *Salmonella*-specificity of CD8^+^ T cell activation. In addition, the observations that CD8^+^ T cells activated by *Salmonella*-infected B cells specifically kill *Salmonella*-infected cells, but do not kill B cells infected with *Staphylococci* or dead *Salmonella*, demonstrate that the CD8^+^ T cell response upon *Salmonella*-infection of autologous B cells is at least in part *Salmonella*-specific.

Various reports have described that B cells play a role in the expansion of *Salmonella*-specific T cells during reinfection, but less in the induction of CD8^+^ T cell responses in primary infection. The reason for this was not understood and has been attributed to the fact that antigen-specific B cells are probably the main B cell population with an antigen presenting function in *Salmonella* infection and that the frequency of antigen-specific B cells is elevated during a secondary infection (reviewed in [32]). Our data now show that indeed the antigen-specific B cells are the cells that are responsible for efficient cross-presentation of *Salmonella* antigens. In addition, our data provide an additional explanation why B cells are mainly involved in the recall response; *Salmonella*-infected B cells do not prime naïve CD8^+^ T cells, but are very efficient in reactivation of cytotoxic CD8^+^ memory T cells. This implies that other APCs (e.g. DCs) are still needed to prime naïve CD8^+^ T cells. This priming by DCs might occur via antigen-presentation after phagocytosis of *Salmonella* itself, or via uptake of apoptotic bodies from other *Salmonella*-infected DCs. In secondary infections however B cells now seem to enter the stage as important APCs for the execution of the CD8^+^ recall response. DCs can still induce CD8^+^ proliferation via direct infection or via suicide cross-presentation upon ingestion of infected apoptotic cells [22], but DCs were shown to mainly activate the T_EM_ compartment [22]. In contrast, *Salmonella*-infected B cells efficiently activate the both the T_CM_ and T_EM_ compartments. This ensures not only the direct terminal differentiation of effector memory cells, but also the expansion of the *Salmonella*-specific CD8^+^ memory T cell compartment, which may both amplify the anti-*Salmonella* immune response and simultaneously ensure generation of anti-*Salmonella* memory for further reinfections.

In summary, we propose a model on the role of B cells in the generation of the humoral and cellular immune response against *Salmonella*. After infection, *Salmonella* enters the body via DCs. The DC-mediated route ensures CD4^+^ T cell activation but is less effective in CD8^+^ T cell activation [30]. The other part of entry for *Salmonella* is via M cells after which it encounters B cells, which are situated in the Peyer's Patches, directly under the M cells. *Salmonella*-specific B cells internalize *Salmonella* via their BCR and are activated. Next, internalization of *Salmonella* leads to presentation of *Salmonella* antigens MHC class II molecules and activation of *Salmonella*-specific CD4^+^ T-helper cells that stimulate *Salmonella*-infected B cells to secrete *Salmonella*-specific antibodies [20], either locally or upon arrival or the infected B cells in the mesenteric lymph node. Upon re-infection, *Salmonella*-specific memory B cells ensure rapid antibody production but *Salmonella*-infected B cells also mediate a strong cytotoxic CD8^+^ recall response to eliminate infected cells. The *Salmonella*-specific CD4^+^ response that aided antibody production in early stages of the immune response is now also required for the activation of the cytotoxic memory T cell response against *Salmonella*. Thus, uptake of *Salmonella* by antigen-specific B cells may generate a survival niche for *Salmonella*, but at the same time it strongly contributes to the generation of effective anti-*Salmonella* immunity at multiple levels of the adaptive immune response.

## Materials and Methods

### Antibodies and fluorophores

mAb anti-human IgM (MH15, Sanquin, Amsterdam, The Netherlands) was mixed with rat anti-mouse IgG1 antibody (RM161.1, Sanquin) and mAb anti-*S. typhimurium* LPS (1E6, Biodesign International, Kennebunk, ME) to generate BCR-LPS tetrameric antibody complexes, used to coat bacteria as previously described [20]. Antagonist anti-human CD40 mouse monoclonal antibody was a kind gift of Dr. L. Boon.

The following labeled anti-human mAbs were obtained from BD Biosciences (San Jose, CA): anti-IFN-γ-FITC, anti-CD27-PE, anti-CD107a-PE, anti-CD8-PerCP-Cy5.5, anti-CD4-APC, anti-CD45RO-PE, AnnexinV-APC and IgG1-PerCP-Cy5.5 isotype control. FITC-conjugated antibody IgG1, IgG2a and IgG2b, IgG1-PE and IgG1-APC isotype controls and PE-conjugated anti-CD8 blocking antibody were obtained from DAKO (Glostrup, Denmark). Anti-CD45RA-FITC and anti-CD45RO-FITC were obtained from Sanquin and DAPI from Sigma-Aldrich (Steinheim, Germany). CFSE (Invitrogen, Paisley, UK) labeling was used in proliferation assays.

### Bacterial growth conditions

GFP expressing-*S. typhimurium* SL1344 was described before [33]. GFP-*Salmonella* defective in SPI-1 (invA mutant) or SPI-2 (ssrA mutant) were a kind gift of M. Rescigno (European Institute of Oncology, Milan, Italy). *Staphylococcus aureus* expressing GFP (RN4220 with pWVW189GFP) was kindly provided by S. A. J. Zaat (Academic Medical Center, Amsterdam, The Netherlands). All bacteria strains were grown overnight at 37°C in Luria-Bertani (LB) broth with carbenicillin or chloramphenicol (Sigma-Aldrich, St Louis, MO) to maintain GFP expression while shaking, subcultured at a dilution of 1∶33 in fresh LB medium and incubated while shaking at 37°C for 3 to 4 hours to obtain exponentially growing bacteria. For coating, bacteria were washed twice with PBS and incubated with BCR-LPS tetrameric antibody complexes for 30 minutes at room temperature and washed twice with PBS to remove unbound antibodies. For experiments with dead *Salmonella*, bacteria were heat killed by incubation at 65°C for 15 minutes.

### Lymphocyte isolation and B lymphocyte infection with *Salmonella*

Human PBMCs were isolated by centrifugation on a Ficoll-Hypaque gradient (Axis-Shield PoC AS, Oslo, Norway) from a buffycoat obtained from healthy donors (Sanquin). All donors provided written informed consent in accordance with the protocol of the local institutional review board, the Medical Ethics Committee of Sanquin Bloodbank (Amsterdam, The Netherlands), and the Medical Ethics Committee of Sanquin approved the study. B and T cells were subsequently purified using anti-CD19, anti-CD4, anti-CD8 Dynabeads and DETACHaBEAD (Invitrogen), according to the manufacturer's instructions. Monocytes were isolated by positive selection using CD14 microbeads and a magnetic cell separator (MACS, Miltenyi Biotec, Bergisch Gladbach, Germany). Monocytes were cultured at a concentration of 1×10^6^ cells/ml in 20 ml Cellgro medium (CellGenix, Freiburg, Germany) supplemented with GM-CSF (1,000 IU/ml; Cellgenix) and IL-4 (800 IU/ml; Cellgenix) in a 80 cm^2^ cell culture flask (Nunc, Roskilde Denmark) to generate immature DCs. At day 6, the DCs were harvested and washed with antibiotic free medium. B lymphocytes or immature DCs were incubated for 45 minutes at 37°C with *Salmonella* without antibiotics. Next, cells were washed to remove unbound bacteria four times and cultured for 1 hour in medium containing 100 µg/ml gentamycin (Invitrogen) to eliminate non-phagocytosed bacteria. Cells were washed and cultured in RPMI 1640 medium w/o phenol red (Lonza, Basel, Switserland), supplemented with 5% FCS (Bodinco, Alkmaar, The Netherlands), 100 U/ml penicillin, 100 µg/ml streptomycin, 2 mM L-Glutamine, 50 µM 2-ME, 20 µg/ml human apo-transferrin ((Sigma-Aldrich), depleted for human IgG with protein G sepharose (Amersham, Uppsala, Sweden)) and 10 µg/ml gentamycin.

### Flow cytometry

Unless described otherwise, 1*10^5^ *Salmonella*-infected cells were cultured with 5*10^4^ CFSE-labeled T cells (CD8^+^ T cells alone or in 1∶1 ratio with CD4^+^ T cells) for 6 days to activate and expand *Salmonella*-specific T cells. 50,000 events were acquired on a LSR II (BD) and analyzed with FACSDiva software (BD). To test plasma membrane markers, 1*10^5^ B cells with 5*10^4^ T cells were cultured for 6 days and after addition of 10 IU/ml IL-2 (Chiron, Emeryville) for another 6 days. All plasma membrane stainings were performed for 15 minutes at room temperature and washed after each incubation with PBS containing 0.1% BSA. 50,000 events were acquired on a LSR II (BD) and analyzed with FACSDiva software (BD). Lymphocytes were gated by forward and side scatter. Dead cells were excluded based on their positive reaction to DAPI staining.

For some experiments, naïve T cells were sorted as CD8^+^CD45RA^+^CD45RO^−^ (T_N_) and memory T cells as CD8^+^CD45RA^−^CD45RO^+^ (T_MEM_), and CD8^+^CD45RO^+^CD27^−^ (T_EM_) and CD8^+^CD45RO^+^CD27^+^ (T_CM_) cells. Populations were >98% purified.

### TCR clonality

CFSE labeled CD8^+^ T cells were stimulated with *Salmonella*-infected B cells or with anti-CD3 (1XE, 1 µg/ml) and anti-CD28 antibodies (CLB.CD28/1, 1 µg/ml). Proliferating and non-proliferating cells were sorted and DNA was isolated using QIAamp DNA Mini Kit, according to the manufacturer's instructions (Qiagen, Valencia, CA). Next, TCR beta clonality was measured using the In Vivo Scribe TCRB Gene Clonality Assay for gel detection, according to manufacturer's instructions (San Diego, CA).

### Intracellular cytokine staining

B cells and T cells were cultured for 12 days, and 10 IU/ml IL-2 was added at day 6 for T cell survival. Cytokine production was measured by intracellular staining after restimulation with 0.1 µg/ml PMA, 1 µg/ml ionomycin and 10 µg/ml brefeldin A (Sigma-Aldrich) for 5 hours. Cells were washed twice with PBS, fixed with 1% formaldehyde (Merck, Darmstadt, Germany) for 15 minutes and after washing twice with PBS, permeabilized with 0.5% saponin (Calbiochem, CA) in PBS containing 1% BSA (Sigma-Aldrich) and incubated with fluorescent antibodies for 30 minutes at room temperature. 50,000 events were acquired on a LSR II (BD) and analyzed with FACSDiva software (BD).

### CD8^+^ degranulation assay

CD8^+^ T cells were primed by 6 day incubation of 1*10^5^ *Salmonella*-infected B cells with 2.5*10^4^ CD4^+^ T cells and 2.5*10^4^ CD8^+^ T cells. The dividing T cells (CD8^+^CFSE^low^) were FACS sorted after 6 days on a MoFlo Sorter (Dakocytomation, Glostrup, Denmark) and cultured with 50 IU/ml IL-2 for 6 more days. Next, isolated autologous B cells were thawed and infected with *Salmonella*. For proteasome inhibition, MG-132 (Sigma-Aldrich) was added at a concentration of 20 µM before incubation with bacteria, and after infection, the cells were cultured overnight in presence of the inhibitor. Next, the cells with proteasome inhibitor were irradiated (30 Gy) to prevent further initiation of antigen presentation after washing the cells to remove the proteasome inhibitor. Cells were not irradiated in other experiments in which no proteasome inhibitor was used. *Salmonella*-infected B cells were incubated in medium containing 10 µg/ml gentamycin for 15 hours at 37°C to allow processing and presentation of *Salmonella* antigens. Subsequently, the B cells were incubated at 37°C for 5 hours together with the primed *Salmonella*-specific CD8^+^ T cells in a ratio of 4∶1, in the presence of anti-CD107a-PE labeled antibodies. Cells were washed twice with wash buffer (1 mM HEPES, 0.15 M NaCl, 5 mM KCl, 1.8 mM CaCl_2_, 1 M MgCl_2_, 0.1% BSA) and stained for CD8 and AnnexinV. After washing twice with washing buffer, DAPI was added and CD8^+^ T cells were analyzed for CD107a expression. 50,000 events were acquired on a LSR II (BD) and analyzed with FACSDiva software (BD).

### ^51^Cr release assay

*Salmonella*-specific CD8^+^ T cells were activated and FACS sorted as described and expanded with 50 IU/ml IL-2. Autologous B cells were thawed and infected with *Salmonella*. After 15h, B cells were labeled with ^51^Cr (185 MBq/ml; Perkin Elmer, Boston, MA) for 45 minutes at 37°C. After washing, the B cells were incubated in a 96-wells U-bottom plate (Costar Corning Inc., NY) with primed CD8^+^ T cells in a 1∶2 ratio. Incubation in medium or in Triton X-100 (1% final concentration; Merck) was used to determine spontaneous and maximum ^51^Cr release, respectively. ^51^Cr release was measured in the supernatant using filters with a gamma counter (Cobra II, Canberra Packard, Mississauga, Canada). The percentage of specific cell lysis was calculated using the following formula:

% specific lysis  =  [experimental release (cpm) – spontaneous release (cpm)]/[maximal release (cpm) – spontaneous release (cpm)] ×100%.

### Statistical analysis

Statistical differences were determined by a paired Student's t test, using GraphPad Prism (version 5.01, GraphPad Software, San Diego, CA).

## Supporting Information

Figure S1 Blocking IL-2 or blocking CD8 reduces *Salmonella*-infected B cell mediated CD8^+^ T cell proliferation. *Salmonella*-infected B cells were cocultured with CFSE labeled CD8^+^ T cells and CD4^+^ T cells, in presence of IL-2 blocking antibodies or CD8 blocking antibodies. CD8^+^ T cell proliferation was measured after 6 days. Data shown are from one representative experiment of two independent experiments with different donors.
(1.38 MB TIF)

Figure S2 Uptake of *Salmonella* by B cells and DCs. B cells (upper panel) or DCs (lower panel) were naturally infected with GFP expressing *Salmonella* (Sal*, left) or infected with anti-IgM coated *Salmonella* (Sal, right). *Salmonella*-GFP positive cells were analyzed 1 hour after infection. Data shown are from one representative experiment of two independent experiments with different donors.
(2.70 MB TIF)
