# Supplementary figures and images for: Antigen-Specific B Cells Reactivate an Effective Cytotoxic T Cell Response against Phagocytosed Salmonella through Cross-Presentation
Source: PLoS One. 2010 Sep 27;5(9):e13016. doi: 10.1371/journal.pone.0013016 (PMC2946406; doi:10.1371/journal.pone.0013016)

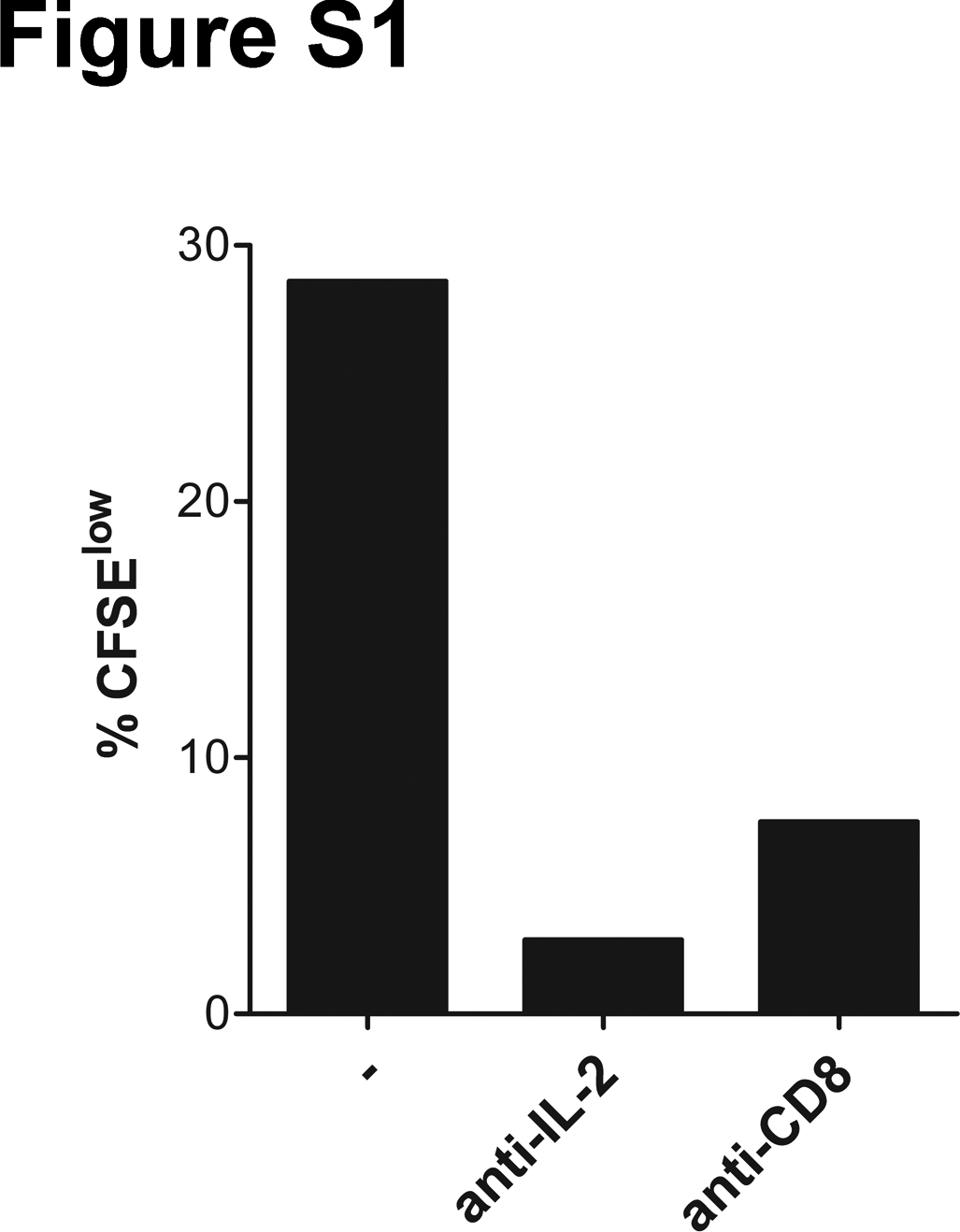

Supplement: Figure S1 — Blocking IL-2 or blocking CD8 reduces Salmonella-infected B cell mediated CD8+ T cell proliferation. Salmonella-infected B cells were cocultured with CFSE labeled CD8+ T cells and CD4+ T cells, in presence of IL-2 blocking antibodies or CD8 blocking antibodies. CD8+ T cell proliferation was measured after 6 days. Data shown are from one representative experiment of two independent experiments with different donors. (1.38 MB TIF) [file pone.0013016.s001.tif]

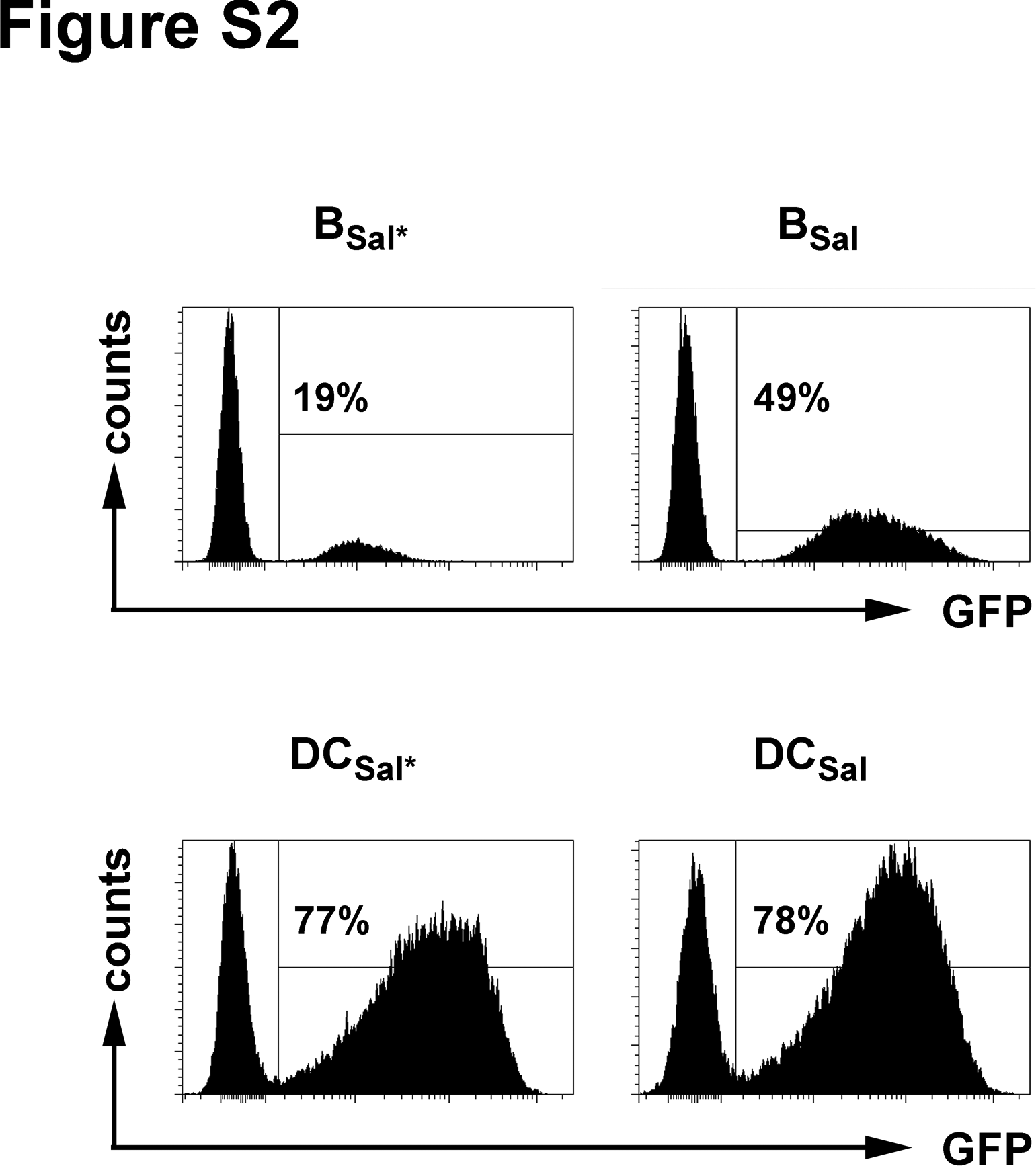

Supplement: Figure S2 — Uptake of Salmonella by B cells and DCs. B cells (upper panel) or DCs (lower panel) were naturally infected with GFP expressing Salmonella (Sal*, left) or infected with anti-IgM coated Salmonella (Sal, right). Salmonella-GFP positive cells were analyzed 1 hour after infection. Data shown are from one representative experiment of two independent experiments with different donors. (2.70 MB TIF) [file pone.0013016.s002.tif]
